# Radiation-Induced Vascular Disease—A State-of-the-Art Review

**DOI:** 10.3389/fcvm.2021.652761

**Published:** 2021-03-30

**Authors:** Eric H. Yang, Konstantinos Marmagkiolis, Dinu V. Balanescu, Abdul Hakeem, Teodora Donisan, William Finch, Renu Virmani, Joerg Herrman, Mehmet Cilingiroglu, Cindy L. Grines, Konstantinos Toutouzas, Cezar Iliescu

**Affiliations:** ^1^Cardio-Oncology Program, Division of Cardiology, Department of Medicine, University of California, Los Angeles, Los Angeles, CA, United States; ^2^Hospital Corporation of America Northside Hospital, St. Petersburg, FL, United States; ^3^Department of Internal Medicine, Beaumont Hospital, Royal Oak, MI, United States; ^4^Division of Cardiovascular Diseases & Hypertension, Robert Wood Johnson Medical School, New Brunswick, NJ, United States; ^5^CVPath Institute, Gaithersburg, MD, United States; ^6^Division of Cardiovascular Diseases, Mayo Clinic, Rochester, MN, United States; ^7^Department of Cardiology, The University of Texas MD Anderson Cancer Center, Houston, TX, United States; ^8^University of Hawaii John Burns School of Medicine, Honolulu, HI, United States; ^9^Cardiovascular Institute, Northside Hospital, Atlanta, GA, United States; ^10^Athens Medical School, Hippokration General Hospital, Athens, Greece

**Keywords:** radiation therapy, cardio-oncology, coronary artery disease, cancer, peripheral arterial disease

## Abstract

Since the 1990s, there has been a steady increase in the number of cancer survivors to an estimated 17 million in 2019 in the US alone. Radiation therapy today is applied to a variety of malignancies and over 50% of cancer patients. The effects of ionizing radiation on cardiac structure and function, so-called radiation-induced heart disease (RIHD), have been extensively studied. We review the available published data on the mechanisms and manifestations of RIHD, with a focus on vascular disease, as well as proposed strategies for its prevention, screening, diagnosis, and management.

## Introduction

Since the 1990s, there has been a steady decline in cancer-related mortality, and consequently an increase in the number of cancer survivors to ~17 million in 2019 in the United States alone (1). Cardiovascular complications from cancer therapy weigh heavily in terms of both morbidity and mortality (2). Among those, radiation-induced cardiovascular disease is one of the most important.

The cardiovascular effects of ionizing radiation were initially observed in atomic bomb survivors and later in patients with therapeutic radiation treatment for medical purposes (3, 4). Radiation therapy (RT) was initially applied to patients with breast cancer and Hodgkin's lymphoma, while today its use has expanded to a variety of malignancies and over 50% of cancer patients (5). The effects of ionizing radiation on cardiac structure and function, so-called radiation-induced heart disease (RIHD), have been extensively studied (6). We review the available published data on the mechanisms and manifestations of RIHD, with a focus on radiation-induced vascular disease (RIVD), as well as proposed strategies for its prevention, screening, diagnosis, and management.

## Pathogenic Mechanisms

**Figure 1:**
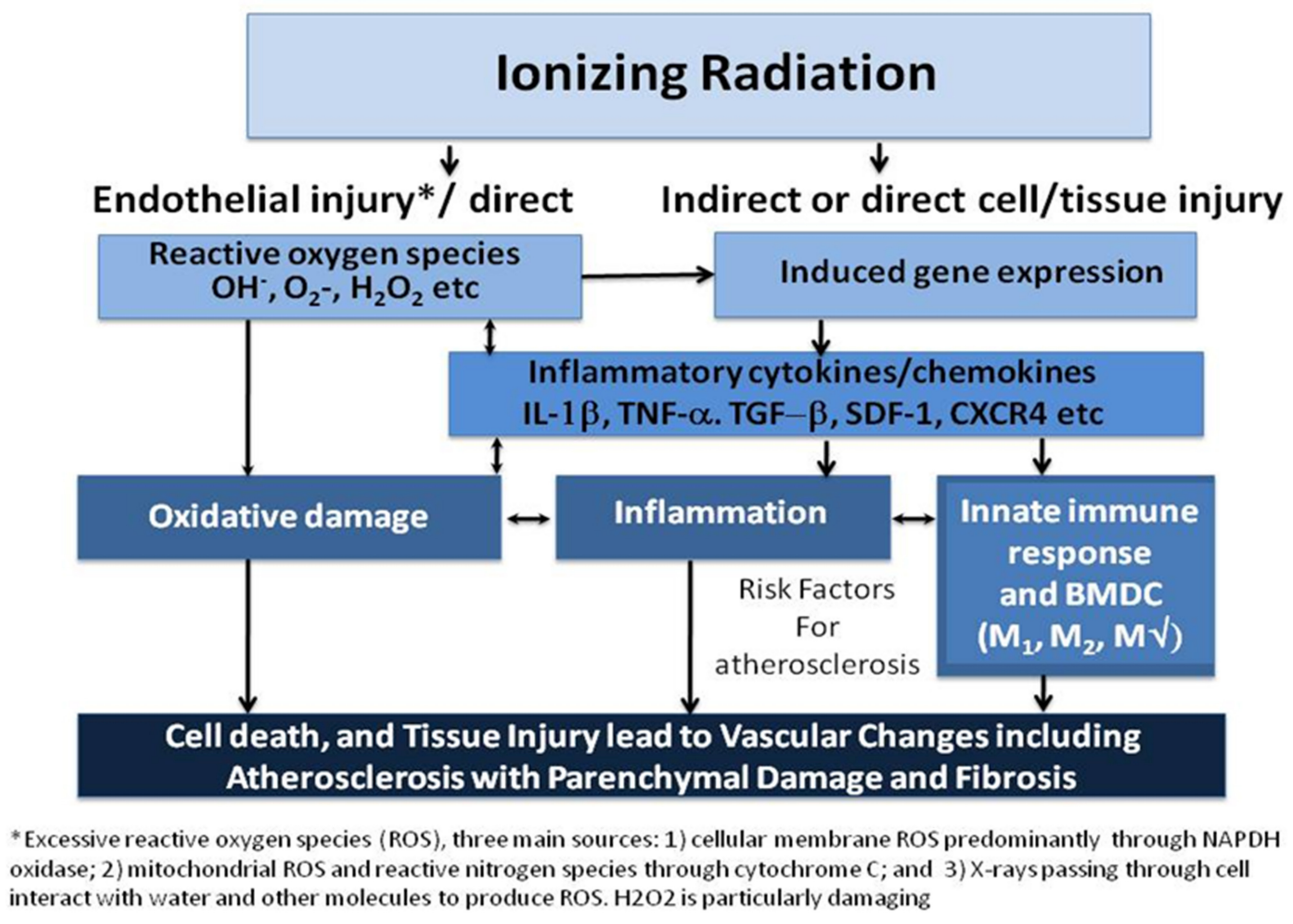
Ionizing radiation causes cell death, both parenchymal and vascular, by multiple mechanisms. Historically, the direct cytotoxicity of radiation was the first identified pathway leading to tissue injury. More recently, another pathway involving inflammation has been identified. A third pathway has been studied in the last few years that implicates the innate immune response including bone marrow-derived cells (BMDC) and both M1, and M2 macrophage (MΦ) in resultant tissue damage. Arrows represent influence of one mechanism on another and suggest potential targets for interfering with the process. Cell death and tissue injury result in accelerated atherosclerosis over 1 to 2 decades that may also result in parenchymal injury to the myocardium and the valves resulting in fibrosis [Modified from (8)].

Ionizing radiation affects not only cancerous, but also non-cancerous cells, especially those that are rapidly proliferating, such as endothelial and bone marrow cells, along with the local parenchymal cells within the radiated territory. Cell cycle arrest, senescence, and apoptosis are induced as a consequence of DNA damage (7) (Figure 1). In high doses, ionizing radiation can result in depletion of parenchymal and vascular endothelial cells, with both macro- and microvascular effects (8).

Oxidative stress due to radiolysis of water molecules into reactive oxygen species promotes endothelial dysfunction and inflammatory changes to the radiation field. Accordingly, radiation induces release of thromboxane and von Willebrand factor and decreased production of prostacyclin, thrombomodulin, and ADPase (9). Von Willebrand factor increases the platelet adhesion to endothelial cells, which may predispose to arterial thrombosis (10). Moreover, degeneration of the vascular smooth muscle, aggregation of foamy histiocytes and adventitial fibrosis have been observed. This is believed to be the precursor of lipid-laden foam cells and the beginning of atherosclerosis formation under the influence of pro-inflammatory cytokine release, such as interleukin(IL)-1, IL-6, tumor necrosis factor (TNF)-α, transforming growth factor-β (TGF-β). Among those, TGF-β is one of the most pleiotropic cytokines, affecting many cellular processes including epithelial cell growth, mesenchymal cell proliferation, and extracellular matrix synthesis (11). Ionizing radiation, even in low doses, induces TGF-β activation, affecting fibroblasts which are transformed into matrix-producing myofibroblasts and leading to fibrosis, a common feature observed in radiation induced heart disease. In individuals with non-small cell lung cancer, when plasma TGF- β1 levels are less than the pretreatment value and <7.5 ng/mL, the chance of radiation induced complications is decreased with higher radiation dose (>73.6 Gy) compared to those in whom the levels are high (12).

**Figure 2:**
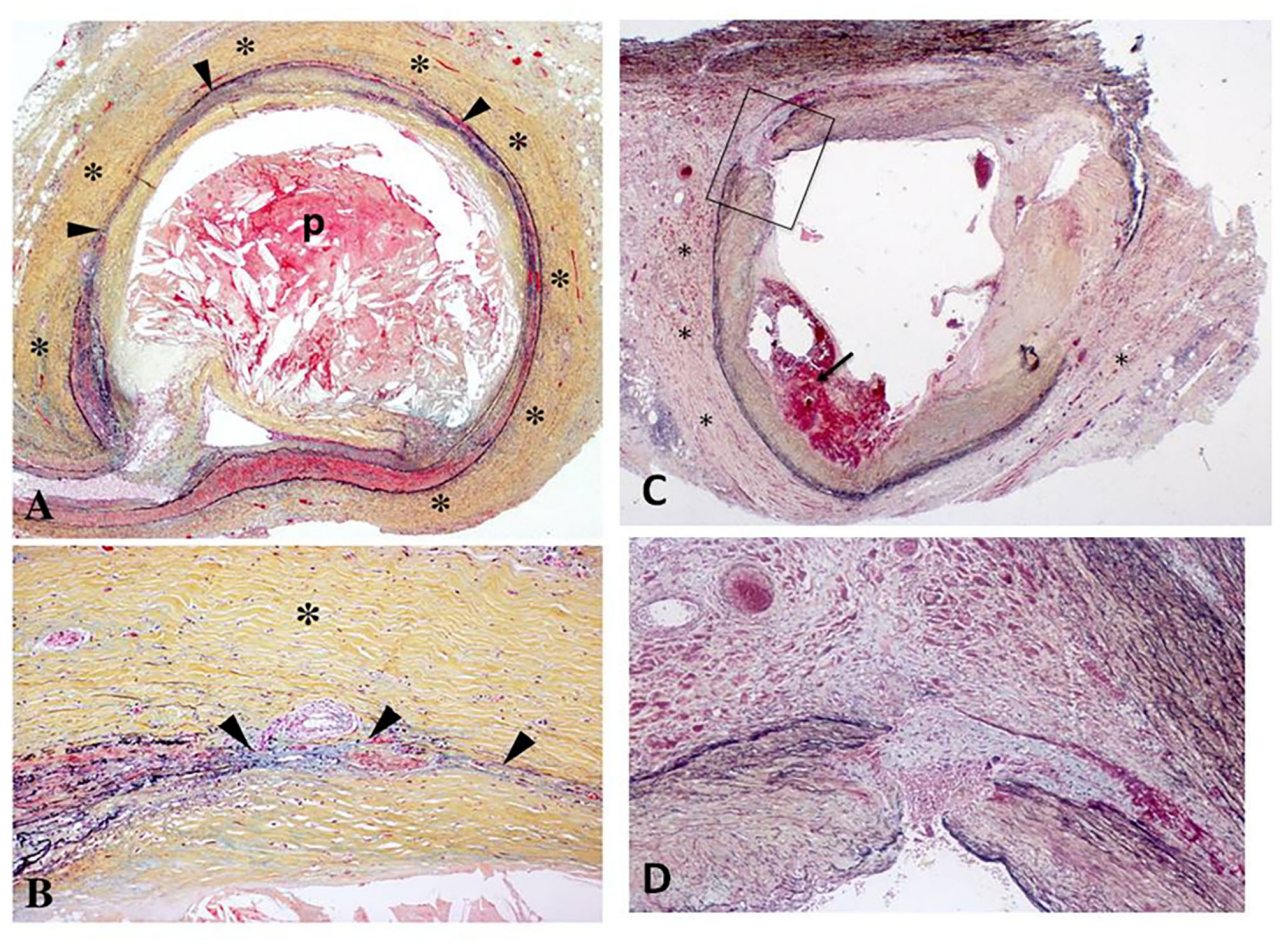
**(A,B)** Histologic section of the left circumflex coronary artery from a 67-year-old patient who received radiation therapy for carcinoma of the lung 7 years prior to sudden death. Low power view **(A)** demonstrates severe adventitial fibrosis (*) and focally extensive destruction of the media (arrowheads) with intimal plaque (p) causing 75% luminal narrowing. The plaque consists mostly of necrotic core that is rich in cholesterol clefts. Note a markedly thickened adventitia (*) at high power **(B)** with medial destruction (arrowheads) [Reproduced with permission from (9)]. **(C,D)** Right coronary artery from a 62-year old man with mediastinal radiation therapy for Hodgkin's disease 25 years antemortem. At autopsy, there was 70% lumen area narrowing **(C)** with intraplaque hemorrhage (arrow), marked adventitial fibrosis (*), and focal destruction of the arterial media (arrowheads). The boxed in area in **(A)** is shown at higher magnification in **(B)**; note medial disruption (arrowheads) and replacement by smooth muscle cells in a collagenous matrix [Reproduced with permission from (14)].

Human pathology studies have described increased intima-media thickness of irradiated arteries, similar to atherosclerotic vascular disease, although medial thinning and adventitial fibrosis were more prominent after irradiation (9, 13) (Figure 2). Intimal lesions following radiation exposure consist primarily of fibrous tissue, while a minority of lesions containing lipid or calcium deposits in addition to fibrosis (15–17). An early finding post-radiation is increased vascular permeability. This is mediated in part by histamine as well as accumulating endothelial cell death. Fibrinogen and von Willebrand factor leak outside the vessels as a result of the increased permeability (18, 19). Fibrinogen is converted to fibrin and evolves into fibrous tissue over time. This permeability is also the likely cause of lipid accumulation and accelerated atherosclerosis in hypercholesterolemic animals (9). In large arteries, damage to the vasa vasorum may contribute to radiation-induced vasculopathy (20). Rarely, arterial ruptures of the aorta, carotid, femoral, or pulmonary arteries have been reported early after massive radiation, although most have argued that it was related to surgery; however, rare case reports describe smooth muscle absence and fraying of elastic fibers (14, 21). In astronauts, spaceflight-associated neuro-ocular syndrome is hypothesized to be caused by radiation-induced angiosclerosis, given the increased radiation exposure during long-duration space flight and on the International Space Station (22).

In addition to intimal fibrosis, the media is often replaced by fibrous tissue, and the adventitia becomes fibrotic. Fibrosis evolves over time and involves all three layers of the vessel wall. Experimental models indicate that cholesterol plaques and thrombosis form within a period of days after radiation exposure (23). Radiation in arteries of hypercholesterolemic animals results in accelerated atherosclerosis (24). The composition, however, is different; the lesions in the aortic roots of irradiated animals are macrophage rich and lipid filled, whereas lesions in non-irradiated ones are collagenous with only minimal macrophage infiltration (25). The plaque burden does not appear to be different with or without radiation.

Pakala et al. recognized a vulnerable plaque phenotype after localized irradiation (26). Another experimental animal study identified increased number of lesions with macrophage-rich cores, low collagen content, and intraplaque hemorrhage in irradiated arteries (13). Intraplaque hemorrhage is known to induce atherosclerosis progression and plaque instability or rupture in human atherosclerotic lesions (27). The effects of one single high radiation dose in the absence of other factors may differ considerably from what is seen in patients who receive multiple cumulative dose fractions. Also, the response to the same radiation dose in different vascular beds may vary for reasons that remain unclear today.

**Figure 3:**
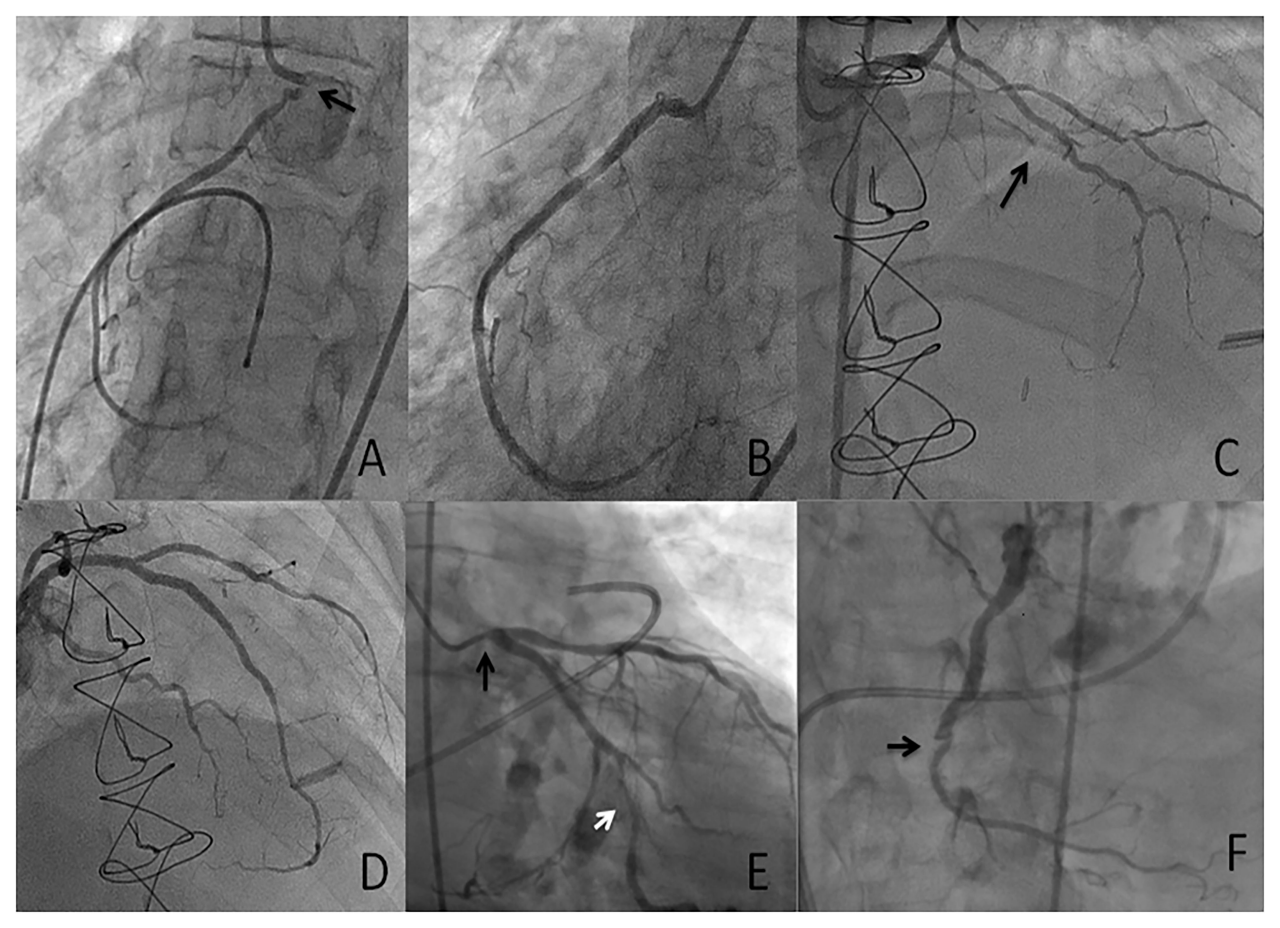
Case presentation 1 **(A,B)** 53-year-old with unstable angina who received mantle radiation 45 years ago for Hodgkin's lymphoma. Severe ostial RCA (arrow) stenosis **(A)** successfully treated with PCI **(B)**. Case presentation 2 **(C,D)** 33-year-old with class III angina who received radiation 7 years ago for thymic carcinoma. Severe diffuse LAD disease (arrow) successfully treated with PCI. Case presentation 3 **(E,F)** 75-year-old with severe symptomatic aortic stenosis who received extensive mantle radiation for esophageal cancer 15 years ago. Diffuse obstructive atherosclerosis involving the ostial left main (**E**, black arrow), the obtuse marginal (OM) branch (**E**, white arrow) and chronic totally occluded right coronary artery \(RCA) (**F**, arrow). The patient was managed with transcatheter aortic valve replacement (TAVR) and PCI of the left main and the OM.

In the coronary circulation the typical pattern associated with radiation-induced coronary artery disease (RICAD) is ostial stenosis with a significantly higher incidence of severe left main disease, followed by ostial right coronary artery and left anterior descending artery stenoses (17). The location and severity directly correlate with the direction and dose of radiation beam (28–30) (Figure 3). Extensive mantle radiation such as for Hodgkin's lymphoma, breast cancer, and esophageal cancer is more likely to cause ostial and multivessel stenoses (23, 28). Conversely, radiation (usually tangential and focal) for breast cancer is more likely to cause focal disease in the mid to distal LAD distribution for left sided breast lesions, whereas involvement of proximal RCA is more common after radiation for right breast lesions) (31, 32).

In the peripheral circulation, vascular toxicity is located in the areas of targeted radiation. The mechanisms of developing PAD relate to vascular damage from ionizing radiation as outlined above. PAD can occur acutely as peri-arterial inflammation, or a chronic process of progressive atherosclerosis and peri-arterial fibrosis (33, 34).

Radiation-associated valvular disease has also been observed in up to ~80% of patients who received chest irradiation, most frequently symptomatic aortic stenosis (35, 36). Surgical aortic valve replacement (SAVR) appears to be associated with worse outcomes in patients who underwent chest irradiation compared to transcatheter aortic valve replacement (TAVR) (37, 38). Bioprosthetic valves, which are increasingly used over mechanical valves (39), are vulnerable to structural valve degeneration (40). The durability of transcatheter bioprostheses appears to be low and similar to surgical bioprostheses, although data are currently limited to at most 10-year follow-up (41). It is currently unclear how these observations relate specifically to patients who underwent chest irradiation, however, several reports suggest that accelerated structural valve degeneration may occur (42, 43). It is unclear whether this effect is due to direct valvular damage (i.e., fibrosis and calcification, as is the case with the native valve), or due to other hematological causes that may also predispose to coronary plaque formation or increased risk of restenosis post-coronary intervention. This hypothesis is further strengthened by the observation that chemotherapy, not only radiation therapy, may predispose to accelerated structural valve degeneration, highlighting the need for further research (43).

## Prevention

Radiation therapy planning aims to minimize the volume of the heart irradiated as well as the radiation dose to the heart. Multiple strategies should be undertaken, including intensity modulated radiation therapy, breath-holding, image guided radiation therapy, and 4-dimensional imaging (44–46). While contemporary approach in radiation oncology has dramatically changed since the initial landmark studies, the impact of such an approach on minimizing cardiovascular risk has not been systematically studied and likely requires long term follow up.

In animal studies, the use of atorvastatin before radiation prevented vascular damage and promoted healing of radioactive injury wound (47, 48). In *in-vitro* studies, pravastatin demonstrated anti-inflammatory and anti-thrombotic effects on irradiated endothelial cells by inhibiting the overproduction of monocyte chemoattractant protein-1, IL-6, and IL-8, and by enhancing the expression of intercellular adhesion molecule-1 (49). Moreover, pravastatin down-regulated the radiation-induced activation of the transcription factor activator protein-1 but not of nuclear factor-kappa-B. In human, pravastatin limits the radiation-induced vascular dysfunction in the skin by decreasing interactions between leukocytes and endothelium and limiting the radiation-induced downregulation of eNOS (50). Finally, an inhibition by pravastatin of increased adhesion of leukocytes and platelets to irradiated endothelial cells was observed. Thus, statins may be considered in therapeutic strategies for the management of patients treated with radiation therapy. However, there are currently no randomized clinical trials that definitively measure the impact of statins on outcomes in RIVD. More robust evidence is required to assess the potential clinical benefit of statins in this setting.

## Screening and Surveillance of RICAD

The most common causes of radiation to mediastinum include treatment for HL and breast cancer. Because of this, a disease of relatively young individuals with very favorable long-term prognosis, CAD, can become a real issue. Up to 3- to 4-fold increase in the risk of myocardial infarction due to coronary artery disease (CAD) has been observed, especially in HL survivors who had mediastinal irradiation or in combination with chemotherapy (51, 52). There is no consensus on the optimal timing at which screening should commence. Some have suggested that screening should be undertaken after 5 years of radiation therapy in patients older than 45 years and between 5 and 10 years for those younger than 45 years. A recent review of non-invasive screening modalities for CAD in HL survivors reported significantly limited diagnostic performance of exercise testing, with a sensitivity of 59% for significant CAD stenosis. Moreover, 25% of those patients subsequently developed symptomatic CAD within a follow-up duration of 6.5 years (53, 54).

Multi-slice CT coronary angiography (CTA) seems an attractive screening modality in this patient subgroup. Recently, high diagnostic accuracy of screening with computed tomographic coronary angiography (CTA) has been shown in asymptomatic patients at intermediate or high risk for CAD (55). Kupeli screened 119 childhood HL survivors of whom only 50% had received mediastinal radiotherapy (median dose 27.5 Gy) after a relatively short median follow-up period of 10 years. Abnormalities on CTA were found in 16%. In a recent phase II trial of asymptomatic HL survivors, the diagnostic accuracy of CTA was evaluated in 48 patients (time since HL diagnosis 21 years). The prevalence of significant CAD (>50% luminal narrowing) on CTA was 20% (*N* = 9). Importantly, stress EKG exhibited very disappointing performance. The two patients with severe left main artery stenosis on CTA and coronary angiography showed no signs of ischemia during the ECG exercise test (56).

**Figure 4:**
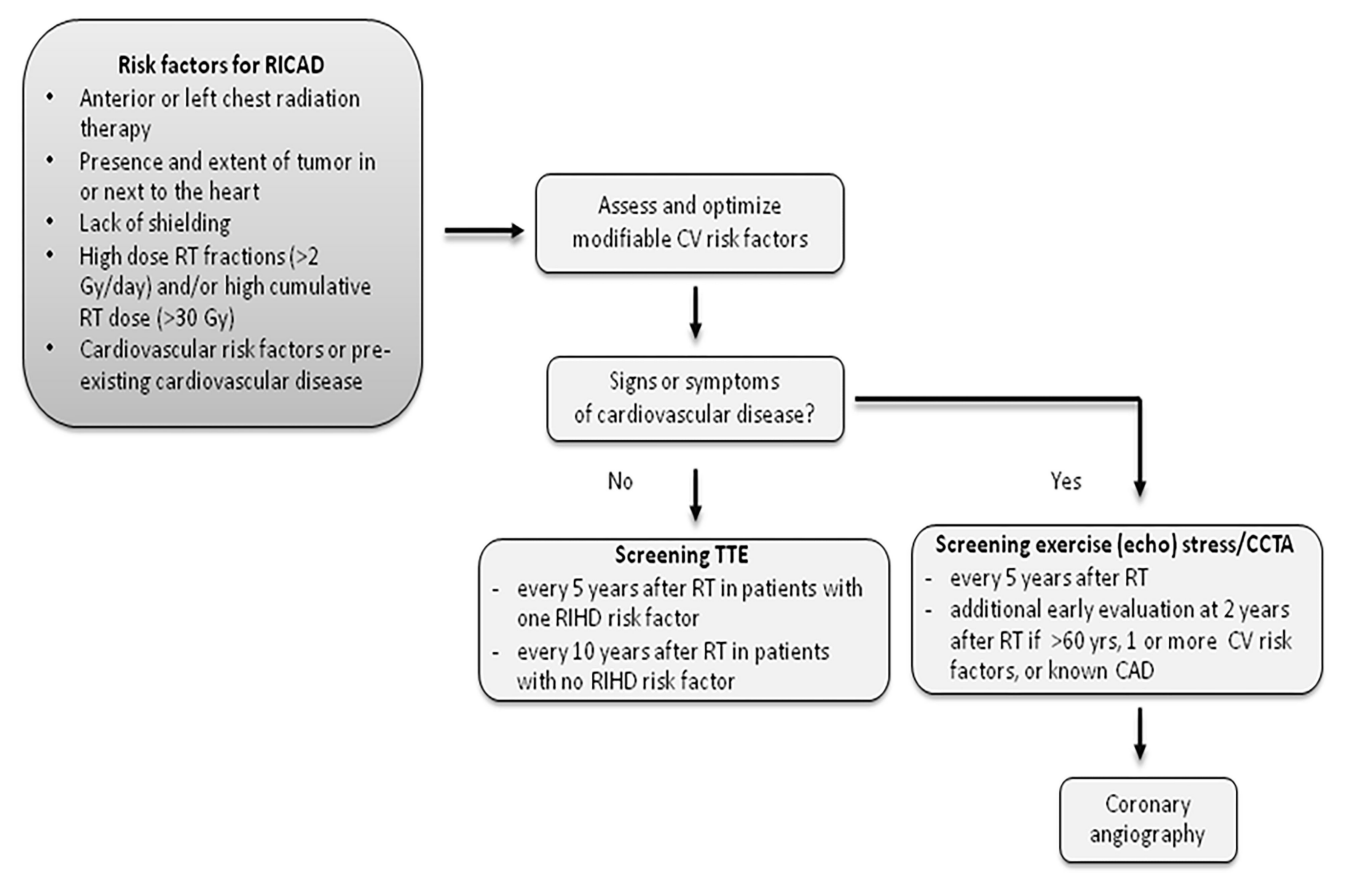
Recommended screening for patients with RICAD. RICAD, radiation-induced coronary artery disease; RT, radiation therapy; CCTA, coronary computed tomography angiography; TTE, transthoracic echocardiogram.

Given the limitations of traditional risk prediction tools and non-invasive modalities, asymptomatic nature of underlying CAD and high risk of subsequent events, patients with prior chest radiation at risk for RICAD must undergo aggressive screening for the presence of underlying CAD. CTA appears to be a very promising test, however, larger studies are needed to confirm the utility of CTA in this population (57) (Figure 4).

## Screening and Surveillance of RIPAD

The timeline as to when to begin screening asymptomatic patients with a history of neck radiotherapy for carotid/subclavian artery disease appears to be dependent on several factors: (1) the type of malignancy (i.e., HL, head and neck malignancies); (2) the age of the patient at the time of treatment; and (3) other cardiovascular risk factors. Although older HL patients who underwent neck radiotherapy appeared to be of higher risk of stroke within a shorter period of time from treatment (around 5 years), patients who were treated at a younger age tended to manifest more subclavian or carotid stenosis after at least a decade of treatment. Retrospective studies of patients who underwent radiation therapy for head and neck malignancies consistently show a significant increase in the risk of cerebrovascular events at 10 years, implying the need for more aggressive, earlier screening in asymptomatic survivors. Suggestions for screening include initial carotid duplex ultrasonography 5 years after radiation treatment, followed by annual ultrasonography, and then tailored to the patient's burden of disease (58–60).

The American Society of Echocardiography, in their 2013 Expert Consensus Statement for patients who have undergone radiation therapy, also mentions carotid artery disease as a long-term sequela of neck irradiation but does not provide recommendations on interval surveillance, other than carotid ultrasound screening in the setting of neurologic symptoms (61). Groarke et al. also suggests screening upon discovering the presence of a carotid bruit or neurologic symptoms and suggests annual surveillance if carotid disease is found that does not warrant intervention; however, data regarding optimal surveillance intervals are lacking (54).

For survivors of childhood/young adult cancer, the Children's Oncology Group Long-term Follow-Up Guidelines recommend that cancer survivors who received >/=40 Gy to the neck region undergo annual neurologic examination and assessment for diminished carotid pulses and/or carotid bruits, with diagnostic imaging of the carotid arteries as recommended. It was also advised to consider a baseline carotid duplex ultrasonography 10 years after radiotherapy. For survivors who received >/=18 Gy of cranial irradiation, an annual neurologic examination is recommended, with brain MRI with diffusion-weighted imaging and MR angiography (62).

For survivors of head and neck tumors, the American Society of Neuroimaging advises screening for carotid artery disease in patients who have received unilateral or bilateral RT at 10 years after treatment. This recommendation was based on studies from patients who received doses >45 Gy, but they acknowledged that no clear relationship has been seen with dose and duration of radiation treatment to validate specific radiation dose information to determine appropriate dose cutoffs for screening. The interval between repeat imaging was unknown, and screening for preexisting carotid artery stenosis prior to radiation treatment was not recommended (63).

More aggressive screening measures are warranted overall in the setting of radiation exposure in the abdomen and lower extremities although the timeline and progression to clinically significant symptoms are less defined. Regardless, patients who experience claudication with a history of abdominal radiation exposure should undergo arterial duplex ultrasound screening and subsequent further imaging (i.e., CTA, MRA) if needed, while the indication to intervene should also be accordance with established guidelines.

## Clinical Manifestations

### Coronary Artery Disease

The latency period between RT and CAD depends on the radiation dose and volume. For doses above 30–35 Gray, RIHD may occur within a year or two of exposure, with the risk increasing with higher radiotherapy dose, younger age at the time of RT, and the presence of traditional atherosclerotic risk factors (51, 52, 64–67). At lower doses, the typical latency period is much longer and is often more than a decade. Survivors of breast cancer and HL are at a greater risk of RICAD as they have a relatively longer cancer specific survival.

In the largest study to date, 2168 breast cancer survivors undergoing radiation therapy were studied in Denmark (64). Patients with major adverse coronary events (myocardial infarction, coronary revascularization and death from ischemic heart disease) were compared to controls. The mean radiation dose to the heart was 4.9 Gy (6.6 Gy for left breast and 2.9 Gy for right breast). There was an exponential relationship between mean radiation dose and major adverse cardiovascular events (MACE). MACE increased by 7.4% for each increase of 1 Gy radiation to the heart with no apparent threshold below which there was no risk. History of previous CAD (relative risk [RR] 6.7), CAD risk factors (RR 1.96), diabetes, smoking and other vascular disease were independently associated with MACE risk. The radiation-related increase in the risk of major coronary events began within the first 5 years after exposure (44% MACE events occurred in the first 10 years of cancer diagnosis, 33% between 10–19 years and 23% 20 years or more) (64).

Similarly, early increases in the risk of myocardial infarction have been reported in studies of patients with HL who received RT, with the risk persisting 25 years and longer. Since HL happens at younger ages and the cumulative radiation dose is higher, the RR of CAD and MACE is proportionately higher compared to older patients (51, 52, 65, 66). The highest risk has been reported in patients aged <25 years at the time of RT, among which the 30-year cumulative rates for any cardiovascular disorder or myocardial infarction are 34.5 and 12.9%, respectively (52). In one large study of 2,232 survivors of disease (mean age 29 years at treatment) the risk of death from heart disease after a mean follow-up of 9.5 years was 3.9% (66). Of the 88 cardiac deaths, 55 were due to myocardial infarction. The average age at death from infarction was 49 years, with 22 deaths in patients <45 years of age. Another large, retrospective single-center study of 415 consecutive patients treated with mantle radiation therapy for HL between 1962 and 1998 found an actuarial incidence of CAD (defined as a history of documented myocardial infarction, coronary artery bypass graft surgery, percutaneous coronary intervention, or more than 75% diameter stenosis on coronary angiography or autopsy) to be 3% at 5 years, 6% at 10 years, and 10% at 20 years. The RR for cardiac death was highest for patients who received a total radiation dose to the chest of >30 Gy and were <20 years of age at the time of their treatment (67). Finally, a Dutch retrospective cohort study of 2,524 Hodgkin's lymphoma patients, with a median age of 27.3 years at the time of diagnosis, showed a significant 40-year cumulative incidence of cardiovascular disease of 50% in patients who had undergone chemotherapy and/or radiotherapy. The presence of mediastinal radiotherapy increased the risks of coronary heart disease (HR 2.7, 95% CI 2.0–3.7), valvular heart disease (HR 6.6, 95% CI 4.0–10.8), and heart failure (HR 2.7, 95%, CI 1.6–4.8) (68).

Patients with RICAD are often younger than those with typical atherosclerotic CAD. In a study evaluating the long-term outcomes of Hodgkin's lymphoma patients by Hancock and colleagues, 69% of patients who suffered a fatal MI because of radiation-induced CAD had no prior symptoms of angina, heart failure or known CAD (69). In older patients, the clinical presentation is similar to atherosclerotic CAD presentation, most often with stable angina (70), acute coronary syndromes or heart failure. Additionally, given the wide spectrum of cardiovascular manifestations from radiation to the heart including pericardial disease (effusion/constriction), valvular heart disease, cardiomyopathies and conduction abnormalities, presence of these should prompt evaluation for suspected RICAD. The location of RT may impact the phenotype of CAD. Tagami et al. demonstrated on a CTA-based study that RT-treated left breast cancer patients were at significantly higher risk of CAD compared to a matched group of right breast cancer patients (28).

The evaluation for suspected CAD in this group of patients should follow the American College of Cardiology guidelines, although atypical symptoms and premature disease should alert the physician to conduct more frequent testing (71). Importantly, as a large proportion of CAD in those patients includes ostial left main and multivessel disease, stress perfusion imaging may result in false negative results on account of balanced ischemia (72). Because of the known limitations of traditional non-invasive functional stress testing, coronary angiography should be considered if there is high clinical suspicion for symptoms, and/or functionally significant disease due to RICAD.

### Radiation-Induced Peripheral Arterial Disease

Peripheral arterial disease (PAD) remains a concern for patients who receive extra-cardiac treatments, although their sequelae and complications are less reported than those of CAD.

#### Stroke and Carotid Artery Disease After Head and Neck Radiation

In patients with head and neck tumors (laryngeal carcinoma, pleomorphic adenoma, and parotid carcinoma), Dorrestejin et al. demonstrated an increased risk (RR 5.6) for ischemic stroke in 367 patients under the age of 60 (median age of 49.3 at the time of treatment) who received RT (dose range 50–66 Gy) as part of their treatment (73). All subtypes of head and neck malignancies were associated with a risk in ischemic stroke, and the RR of stroke also increased with concurrent risk factors such as diabetes and hypertension. In Dorrestejin's study, the 10-year relative risk for a CVA event was 10.1 (95% CI 4.4–20.0), and the 15-year cumulative risk 12.0% (95% CI 6.5–21.4%). Radiation for head and neck tumors often utilizes a higher dose than that for HL, with a mean of 60.6 Gy in one study, and may be as high as 70–80 Gy (59, 74). Dorrestejin et al.'s patient population had received a range of doses from 50 to 66 Gy, while another study with a dose range of 40–50 Gy did not find a statistically significant increase in stroke (75, 76). Haynes et al. did not find a dose effect for ischemic stroke in patients with history of head and neck irradiation, however follow-up was only 2 years (much shorter than the observed time to symptoms after radiation), and the dose range (59.4–76.8 Gy) was likely too narrow to detect a statistical effect. Seventy-one patients with nasopharyngeal carcinoma (mean age of 53.6 years) with a history of RT (mean dose of 56.4 Gy) were compared with a control group which showed an increased prevalence of 79% for carotid artery stenosis as diagnosed by duplex carotid ultrasound compared to 21% in the control group who had similar cardiovascular risk factors. Time to diagnosis from treatment ranged from 4 to 20 years (77).

An analysis of 6,862 patients greater than the age of 65 from the Surveillance, Epidemiology and End Results (SEER)-Medicare cohort who were diagnosed with non-metastatic head and neck cancer between 1992 and 2002 found a higher 10-year incidence of cerebrovascular events in patients treated with RT alone vs. those treated with surgery and RT, and those treated with surgery alone (34% vs. 25% vs. 26%, *p* < 0.001). No difference was found for surgery plus RT vs. surgery alone, and patients with RT had no increased cardiac risk compared to other treatment groups (78).

Data regarding the contribution of RT toward stroke risk in patients undergoing RT for pituitary adenomas is conflicting, although it overall appears to be higher compared to age-matched controls, regardless of the use of adjuvant RT. In a total of 462 patients with pituitary adenomas undergoing surgery with or without RT (median age of 46 years in the RT cohort), a higher incidence of stroke was seen compared with the general population after a median time of 9 years of follow-up. However, there was no association of increased risk of stroke with postoperative radiotherapy (median of 45 Gy). Major stroke risk factors included preexisting coronary and/or peripheral vascular disease and hypertension (79). However, different findings were seen in another study of 806 patients (mean age of 48.3 years with mean follow up time of 10.0 years) with the RT arm (*n* = 456) receiving a mean dose of 46.2 Gy. A higher incidence of cerebrovascular events was seen in men who underwent RT compared to those who did not (hazard ratio 2.99, 95% CI 1.31–6.79); no significant difference was seen in women who underwent RT. There was also no association of RT with mortality (80).

In adult survivors of childhood/young adult cancers, an analysis of the Childhood Cancer Survivor Study (CCSS), a cohort of long-term childhood cancer survivors diagnosed between 1970 and 1986, revealed an overall 10-fold higher relative risk for stroke in the cohort subjects compared to their siblings as controls. Within the CCSS, 27.4% reported history of brain radiation and up to 21.8% history of chest irradiation. Conditions with elevated long-term stroke risks were acute lymphoblastic leukemia (ALL), brain tumors, and Hodgkin's lymphoma (81). Mean cranial radiation doses of >/= 30 Gy were associated with an increased stroke risk in both leukemia and brain tumor survivors in a dose-dependent fashion (82). Within the CCSS, Hodgkin's disease patients who suffered a cerebrovascular event received a median mantle radiation dose of 40 Gy (82).

In patients who have received cranial radiation for disease states such as brain tumors, HL, and/or leukemia, the incidence of vascular related sequelae is not well-defined. For primary brain tumors located in the suprasellar region, high doses of RT (>/= 45 Gy) may be needed for effective treatment doses. However, a variety of cerebrovascular sequelae have been described, such as narrowing of the internal carotid arteries to form a moyamoya-like state, leading collateral blood vessel formation to supply flow to hypoperfused areas of the brain. Vascular malformations can also develop, including venous based cavernous malformations, aneurysms, and telangiectasias. Intracranial aneurysms are rare complications from radiotherapy but can be life-threatening. Small vessel vasculopathy can also develop, that can lead to calcification of the basal ganglia, leading to symptoms such as complicated migraine-like symptoms that can also present with stroke-like findings; this finding is referred to as Stroke-Like Migraine after Radiation Therapy (SMART) syndrome (83, 84).

For glottis tumors requiring neck radiotherapy, an analysis of 1,413 patients who were >66 years of age showed a high 10-year risk of cerebrovascular disease of up to 56.5% vs. 48.7% who received surgery alone without radiation, which was not statistically significant between the two groups but showed an overall high rate of cerebrovascular events likely due to preexisting comorbidities (85). The elapsed time interval after radiation is the strongest predictor of cerebrovascular events (86).

#### Supraclavicular and Mediastinal Radiation

For patients with a history of supraclavicular and mediastinal radiation, several malignancies have been associated with a higher risk of cerebrovascular events and carotid artery disease, particularly lymphoma and head and neck malignancies. A retrospective analysis of 415 patients with a history of mantle field radiation for HL showed a 7.4% prevalence of carotid and/or subclavian artery disease after a median follow-up time of 17 years. For those who suffered a TIA or stroke, the median age when undergoing radiotherapy was 51 years and the median time from therapy to event was 5.6 years (67). On the contrary, the median age of patients with isolated subclavian or carotid artery stenosis was 20 years at the time of therapy and the median time from therapy to event was 21 years. The median cumulative low-cervical radiation dose for patients who developed subclavian stenosis and carotid artery disease was 44 and 38 Gy, respectively (67). Another retrospective study of 2,201 survivors of HL treated with mantle field radiation therapy before the age of 51 years showed at a median follow up of 17.5 years an incidence ratio of stroke of 2.2 (95% CI 1.7–2.8) and 3.1 for TIA (95% CI 2.2–4.2). Radiation to the neck and mediastinum was an independent risk factor for ischemic cerebrovascular disease (HR 2.5, 95% CI 1.1–5.6) 30 years after treatment in addition to hypertension, diabetes and hypercholesteremia while obesity and smoking were not (87).

A retrospective analysis of radiation induced carotid artery disease from mostly laryngeal/nasopharyngeal cancer and lymphoma survivors showed a higher incidence of plaque that was ulcerative, mobile, and vulnerable by MRI and ultrasound imaging compared to control subjects who did not receive radiation. There was also a higher cerebrovascular event rate in patients who underwent carotid artery stenting (CAS) vs. carotid endarterectomy (CEA) (88). Another prospective cohort studied 42 patients (mean age 53 years) with a history of head and neck cancer survivors who underwent radiotherapy (mean dose to common carotid and internal carotid arteries of 57 and 61 Gy, respectively) and underwent carotid MRA imaging at a mean follow up of 6.8 years. Significantly more vessel wall thickening (>/=2 mm) was seen in irradiated vs. non-irradiated carotid arterial segments (58% vs 27% in common carotid arteries, 24% vs. 6% of internal carotid arteries, *p* < 0.05) with no difference in signal intensities of the vessel walls (89). An overall meta-analysis of case-control studies and randomized clinical trials on neck-directed radiation-induced disease of the extracranial carotid arteries demonstrated a statistically significant difference in overall stenosis rate in patients who received radiotherapy than controls, with a pooled risk ratio of 4.38 (95% CI 2.98–6.45, *p* < 0.00001) and severe stenosis with a pooled risk ratio of 7.51 (95% CI 2.78–20.32, *p* < 0.0001) (90).

Symptomatic axillary artery stenosis requiring percutaneous intervention and manifesting more than 10 years after radiation therapy for breast cancer has also been reported (91).

#### Abdominal and Pelvic Radiation

RIPAD has been reported in patients who received abdominal radiation for lymphoma (92, 93), abdominal sarcomas (94), as well as for genitourinary malignancies (95). Clinical presentations have ranged from acute thrombotic occlusion to chronic claudication. Radiation induced renovascular hypertension has been reported in HL (92, 93), and severe iliac peripheral vascular disease has been documented in patients who received RT for cervical cancer with preoperative external radiotherapy ranging from 40 to 45 Gys (not including vaginal brachytherapy) presenting anywhere from 1 to 47 years after exposure (95).

### Radiation-Induced Venous and Lymphatic Disease

There are few data on radiation-induced venous and lymphatic disease, mostly limited to case reports. Radiation-associated venous endothelial injury predisposing to thrombosis has been hypothesized, with few case reports describing upper extremity deep venous thrombosis years after chest irradiation (96–98). However, a causal relationship is difficult to demonstrate, as cancer patients and survivors are already at increased risk of vascular thrombosis due to the pro-thrombotic state of malignancy, independently of radiation therapy. Venous stenosis imposing endovascular stenting, as well as fibrosis without thrombosis, have also rarely been described (99–101).

Lymphedema is a well-described complication of radiation therapy. However, there are few data on the mechanisms and long-term clinical implications of this complication. Histologic studies suggest that radiation induces a loss of capillary lymphatics and a dose-dependent increase in lymphatic endothelial cells apoptosis, leading to delayed fibrosis (102, 103). Damage to the pulmonary lymphatic vasculature has been described even after a single dose of radiation, which may lead to delayed lung repair (103).

## Treatment Considerations for RICAD

Medical treatment for RICAD should follow the same secondary prevention strategies that are recommended for traditional atherosclerotic CAD per ACC/AHA guidelines, including aspirin 81 mg per day (in the absence of contraindications), lifestyle interventions and pharmacotherapies to achieve target LDL, blood pressure and blood sugar goals (104). While there is significant paucity of data on the role of preventive therapies in patients with RICAD, it only seems intuitive to aggressively institute preventive measures in patients at risk for RICAD. Long term, prospective trials are needed in looking at the primary prevention impact of aforementioned pharmacologic strategies of patients exposed to radiotherapy. For symptomatic patients and asymptomatic patients with high risk anatomy (and or large ischemic burden) revascularization should be undertaken.

### Role of Percutaneous Coronary Intervention

The role of percutaneous coronary intervention (PCI) with drug eluting stents (DES) may be a viable and potentially durable revascularization strategy for flow limiting RICAD (105–107). However, there have been few studies reporting outcomes (108–111).

In one of the earlier studies, 15 lymphoma patients with RICAD undergoing bare metal stent (BMS) implantation were compared to 7 lymphoma patients without previous radiation and over 12,000 controls undergoing BMS implantation (108). On follow up angiography at 6 months, the authors noted a very high rate of angiographic in-stent restenosis (>50% diameter stenosis) in the RICAD arm compared to others (85.6% vs. 17% vs. 25%). Two thirds (66%) of patients in the RICAD arm underwent repeat PCI compared to 14% and 16% in the other two groups, respectively. Importantly, there were no adverse events in the RICAD group within the first 30 days. At 1 year, there was no mortality reported in the RICAD group vs. 4.4% in the control arm. This study demonstrated a very high incidence of angiographic restenosis with the use of BMS in RICAD patients (108). It is unclear how many of these were physiologically significant and clinically relevant as there was only one myocardial infarction at 1 year in the RICAD group. DES may be the preferred modality in such patients, although clinical data are lacking.

In another case control study comparing 41 patients with RICAD (68% breast cancer treated) with 82 control patients showed an excess of all cause (39% vs. 12%) and cardiac mortality (12% vs. 3.7%) in RICAD patients compared to controls at a mean follow up of 5+/−2 years after stenting (80% BMS). There was no difference in acute myocardial infarction (4.9% vs. 3.7%) during the follow up period (109).

The effect of more recent radiation before stenting, or radiation after stenting is unknown. Liang et al. (110) studied 115 patients treated with EBRT (external beam radiation therapy) for a median of 3.6 years after stenting (group A) and 45 patients treated with EBRT a median 2.2 years before stenting (group B), demonstrating that long-term mean target lesion revascularization rates in group A (3.2 vs. 6.6%; *p* = 0.31) and group B (9.2 vs. 9.7%; *p* = 0.79) were similar to rates in corresponding control patients (group A: 1,390 control patients; group B: 439 control patients). The authors concluded that thoracic EBRT is not associated with increased stent failure rates when used a few years before or after PCI, and a history of PCI should not preclude the use of curative thoracic EBRT in cancer patients or vice versa. Sixty percent of patients in group B had DES. Given the median duration of 2.2 years after EBRT, it remains unclear, if this was indeed RICAD. Nevertheless, these results do provide some reassurance that radiation therapy in itself does not increase the risk of stent failure (although data are not available for early radiation after stenting) and PCI could be considered as a viable revascularization option.

Endovascular treatment for radiation-induced venous stenosis has also been reported, although data are limited to case reports (101).

### Role of Surgical Revascularization

Cardiac surgery in the previously radiated thorax is associated with higher rates of post-operative complications, as well as short- and long-term mortality (32, 112, 113). Major contributing factors include extensive scar tissue and adhesions around the heart, lungs and pericardium from previous radiation that make isolation and harvesting of grafts difficult, fragility and disease of LIMA, presence of concomitant valvular lesions, pericardial constriction (often requiring concomitant corrective surgeries at the time of CABG), left ventricular dysfunction and poor pulmonary reserve (114–121). Importantly, surgeons have historically shied away from using LIMA grafts, although the evidence for such practice is conflicting (119, 120). Furthermore, the discrepancy in the use of LIMA as well as surgical outcomes are variable depending on the extent of previous thoracic radiation (mortality (in hospital and at 4 years) 2.4%/20% in tangential/limited radiation (breast cancer) vs. 13%/43% in extensively radiated patients (HL/thymoma) (122). Studies reporting favorable outcomes with use of LIMA predominantly enrolled patients with previous breast surgery (limited tangential radiation) (121). If surgical revascularization is considered for multivessel RICAD, then angiography of the LIMA and/or RIMA should be done in mediastinal radiation patients to ensure patency of these vessels as potential graft conduits.

A recent study from the Cleveland Clinic, evaluated 173 patients with radiation heart disease (75% women; age, 63 ± 14 years) undergoing cardiac surgery (largest cohort to date) and 305 comparison patients (74% women; age, 63 ± 14 years) (123). In the RT group, the vast majority had prior breast cancer (53%) and HL (27%), and the mean time from radiation was 18 ± 12 years. Only one third of patients in either group had isolated single-valve or coronary bypass procedure (only 15% patients underwent isolated CABG); the rest were combination procedures (CABG with valve replacements/repair). During a mean follow-up of 7.6 ± 3 years, a significantly higher proportion of patients in the radiation group died compared to controls (55% vs. 28%; log-rank *P* < 0.001). Furthermore, even in patients undergoing isolated CABG mortality was significantly higher compared to controls (46% vs. 28%). On multivariable Cox proportional hazard analysis, the presence of radiation heart disease (hazard ratio, 2.47; 95% confidence interval, 1.82–3.36), increasing EuroSCORE (hazard ratio, 1.22; 95% confidence interval, 1.16–1.29), and lack of β-blockers (hazard ratio, 0.66; 95% confidence interval, 0.47–0.93) were associated with increased mortality (all *p* < 0.01). Based on these findings, the authors recommended alternate approaches to RICAD including percutaneous coronary and or valvular approaches.

## Clinical Decision Making

Generally, patients with RICAD should undergo PCI per ACC/AHA guidelines and appropriateness criteria. Given the proximal location of RICAD lesions and the high risk of stent failure with BMS, DES are preferred. For patients with complex RICAD, a multidisciplinary approach involving the “heart team” and oncologist is important for optimal clinical decision making. Depending on local surgical and interventional expertise, surgical risk patients may be amenable to percutaneous or even hybrid approaches. Totally percutaneous approaches for valvular heart disease and CAD may be appropriate. Isolated LM disease has comparable or even superior outcomes than CABG. Current guidelines provide a Class IIa (Level of Evidence B) recommendation for PCI of left main ostial or shaft disease when it exists in isolation or in combination with 1-vessel disease. Our team has recently published an expert consensus statement regarding special consideration of cardio-oncology patients in the cardiac catheterization laboratory (124).

### Treatment Considerations for RIPAD

In the era of percutaneous approaches with distal embolization protection showing favorable outcomes compared to surgical intervention for significant carotid artery disease with regards to MACE (CREST, SAPPHIRE, Gurm-SAPPHIRE), multiple case series have shown favorable outcomes in percutaneous carotid artery stenting for radiation induced carotid artery stenosis (125–128). However, these studies were small and localized to specific institutions, where the level of competency may vary. Carotid stenting in several case series have shown low rates of stroke—Al-Mubarak et al. reported that 1 in 14 patients had a stroke post-stenting (129) and Ting et al. reported 1 stroke out of 16 patients that later led to death (126). A meta-analysis comparing carotid artery stenting and carotid endarterectomy (CEA) for radiation induced carotid artery disease showed similar pooled rates of perioperative cerebrovascular events (3.9% for stenting vs. 3.5% for endarterectomy), although late outcomes favored CEA. There was a higher risk for cranial nerve injury after CEA but higher rate of restenosis after carotid artery stenting (130). The major limitation of this review was the lack of randomized studies, as well as variation in patient selection and small sample sizes. A recent study comparing CAS in patients with radiation therapy-associated carotid stenosis showed similar composite 30-day stroke, myocardial infarction, and mortality (XRT: 2.6% vs. non-XRT: 3.9%; P = NS.) and 50% restenosis rates (XRT: 9.4% vs. non-XRT: 8.6%; P = NS) compared to CAS performed in patients with no radiation therapy (131). While a randomized trial comparing the two strategies is warranted, ultimately the individual institutional experience must be put into account when determining the most optimal interventional strategy for radiation induced carotid artery disease, and should overall be in accordance with ACC/AHA/SCAI guidelines.

For RIPAD, because of the concern of concurrent accelerated fibrosis and associated elastic recoil has led to the idea that stenting as opposed to percutaneous angioplasty alone is more effective, particularly for iliofemoral disease from RT (94, 132). Percutaneous transluminal angioplasty with or without stent placement was performed with success in radiation induced renal artery stenosis (92, 93). Lower extremity bypass has also been employed showing efficacy in small case series and case reports (94, 95). However, data is overall extremely limited due to the overall lack of peripheral vascular disease related cases in the literature, and this may represent underreporting.

Medical management for patients with significant risk factors (as with cardiovascular disease) should be on aggressive antiplatelet and statin therapy as well as antihypertensive therapy as needed. It is essential for each institution to weigh the risks and benefits when determining surgical vs. percutaneous approaches in a multidisciplinary fashion amongst interventional cardiologists, interventional radiologists, and vascular surgeons. In the absence of randomized controlled trials, recommendations regarding surgical vs. percutaneous management of RIPAD should be individualized based on the “vascular team” consensus.

## Conclusion

As the population of cancer survivors is increasing with more effective cancer therapies, RIHD emerged as an important component of radiation cardiotoxicity. RICAD and RIPAD should be screened, diagnosed, and promptly managed to assure better quality of life and improved survival rates. Collaboration between cardiologists and hematologists/oncologists is of prime importance. Most data on RIVD is derived from case series and single-center studies vulnerable to selection bias, from institutions with different strategies and levels of experience in addressing RIVD. The decision of endovascular vs. surgical management of RIPAD should be individualized based on patient factors, as well as institutional experience. Further research via focused randomized controlled trials is needed to determine the optimal prevention, screening, and management methods.

## Author Contributions

All authors listed have made a substantial, direct and intellectual contribution to the work, and approved it for publication.

## Conflict of Interest

The authors declare that the research was conducted in the absence of any commercial or financial relationships that could be construed as a potential conflict of interest.
